# Association Between Dyslipidemia and Lichen Planus: A Cross-Sectional Study in a Tertiary Care Hospital

**DOI:** 10.7759/cureus.82401

**Published:** 2025-04-16

**Authors:** Jeyraveena N. M., Manu Vidhya H., Murugan Sundaram, Sudha Rangarajan, Adikrishnan Swaminathan

**Affiliations:** 1 Dermatology, Sri Ramachandra Institute of Higher Education and Research, Chennai, IND

**Keywords:** cardiovascular risk, comorbidities, dyslipidemia, inflammatory disorders, lichen planus, lipid profile

## Abstract

Background

Lichen planus (LP) is an immune-mediated inflammatory disorder of the skin, mucous membranes, and nails. Several studies have reported a potential association between lichen planus and dyslipidemia, suggesting a possible link between this chronic inflammatory condition and metabolic disturbances. However, existing evidence has shown variable results across different populations. This study was conducted to further investigate the association between lichen planus and dyslipidemia.

Methods

A comparative cross-sectional study was conducted at Sri Ramachandra Institute of Higher Education and Research, Chennai, from August 2022 to August 2024. The study included 67 clinically diagnosed LP patients and 67 age- and sex-matched healthy controls. Detailed clinical examinations and fasting lipid profiles were performed. Statistical tools such as descriptive statistics, chi-square tests, and paired t-tests were applied.

Results

Patients with lichen planus (LP) exhibited statistically significant higher total cholesterol (p=0.001) and low-density lipoproteins (LDL) (p=0.001) levels compared to controls in both males and females. However, significantly elevated triglyceride levels were observed only in male LP patients (p=0.04). The high-density lipoproteins (HDL) (p=0.534) levels were similar between both groups. The prevalence of hypertension, diabetes, hypothyroidism, and cardiovascular disease was also higher in LP patients. Duration of illness was positively correlated with dyslipidemia prevalence.

Conclusion

LP patients exhibit higher lipid levels and comorbidities, highlighting the need for comprehensive management strategies such as routine lipid profile monitoring, early intervention, and lifestyle modification counselling, to mitigate the risk of dyslipidemia to address cardiovascular risk factors.

## Introduction

Lichen planus (LP) is a chronic inflammatory disease of mucous membranes, skin, hair, and nails. The classic cutaneous manifestation is flat-topped, violaceous, pruritic, polygonal papules that are often seen bilateral and symmetrical most commonly occurring on the extremities [1]. The pathogenesis involves antigens processed by Langerhans cells presented to T lymphocytes, triggering cytokine release, including tumour necrosis factor-alpha (TNF-α), IL-6, IL-10, and IL-4. These cytokines modulate the acute phase reactants, known to alter lipid levels, which play an important role in eliminating the inflammatory response and help in tissue regeneration [2]. When the inflammation becomes chronic, the lipid derangements are sustained, causing more harm.

In lichen planus, there is also substantial disruption of antioxidant mechanisms, leading to oxidative stress, resulting in protein, DNA, and lipid damage [1]. It is well recognised that oxidative stress and dyslipidemia, when present chronically, increase the likelihood of cardiovascular disease. This risk increases in the presence of other comorbidities such as hypertension, diabetes mellitus, and metabolic syndrome [3]. A meta-analysis published by YC Lai et al, found that patients with LP were 75% more likely to have dyslipidemia [4]. Hence, we conducted this study to further investigate the association between lichen planus and dyslipidemia, as early detection helps in the prevention and management of dyslipidemia and in preventing the associated complications.

## Materials and methods

This was a comparative cross-sectional study conducted in the Department of Dermatology, Venereology, and Leprosy for a period of 2 years (August 2022 to August 2024) at Sri Ramachandra Institute of Higher Education and Research after obtaining institutional ethical committee approval (CSP-MED/22/JUL/77/76).

A total of 134 patients were enrolled in the study, with 67 patients in the lichen planus group and 67 in the control group (patients without lichen planus). After obtaining informed consent, patients aged above 18 years, both male and female, clinically diagnosed with lichen planus, and healthy controls matched for age and sex with the lichen planus patients, who were willing to undergo the required investigations were enrolled in the study. Patients and controls who were pregnant and/or on systemic steroids, retinoids, and a history of medications on lipid-lowering drugs or any medications affecting lipid metabolism for at least 1 month prior to participation in the study were excluded.

A detailed history-taking and clinical examination were done and the details were recorded along with patients' demographic parameters and BMI. The fasting lipid profile was done for the patients and controls after 8 to 12 hours of fasting. A patient is considered to have dyslipidemia if one of the following is present according to the United States National Cholesterol Education Program Expert Panel Adult Treatment Panel III (NCEP ATP III). Serum total cholesterol >200 mg/dl, serum triglycerides >150 mg/dl, serum HDL <40 mg/dl, serum LDL >130 mg/dl or on treatment for dyslipidemia [5].

Statistical analysis

Descriptive statistics were presented for the data - categorical variables were presented as numbers and percentages, while numerical variables were presented as means and standard deviations. The rates in the groups were compared with Chi-square tests and unpaired t-tests (independent sample t-test). The statistical alpha significance level was accepted as p<0.05. IBM SPSS version 26.0 (IBM Corp., Armonk, USA) was utilized for statistical analysis.

## Results

A total of 67 patients and 67 controls were included in the study. Most patients were in the age group of 30 to 40 years. The majority of patients included in the study were females with a female-to-male ratio of 1:0.8. Most patients sought medical attention within two months of symptom onset. The average duration of their illness was 6.35 months. BMI calculated as per Asian classification showed that the majority of the patients were pre-obese, 26 (38.8%), whereas the majority of the controls had a normal BMI, 27 (40.3%), with no statistical significance. There was no notable difference in personal habits between the groups. The prevalence of diabetes mellitus, hypertension, and hypothyroidism was slightly higher in the lichen planus patients than in the controls but was not statistically significant (Table 1).

**Table 1:** Comparison of demographic characteristics between the patient and control groups

Parameter	Patient group (n=67)	Control group (n=67)	Statistical test	Statistical test value	p-value
Age					
Mean (min-max)	40.75 (18-80)	43.4 (19-76)	Unpaired t-test	0.401	0.086
Gender, n (%)					
Female	36 (53.7)	36 (53.7)	Chi-square test	20.403	1.0
Male	31 (46.3)	31 (46.3)			
Socioeconomic status, n (%)					
Lower	5 (7.4)	8 (12.1)	Chi-square test	32.842	0.884
Upper lower	27 (40.2)	32 (47.7)			
Lower middle	35 (52.4)	27 (40.2)			
BMI according to Asian classification, n (%)					
Underweight	0	1 (1.5)	Chi-square test	12.758	0.659
Normal	23 (34.3)	27 (40.3)			
Overweight	12 (17.9)	10 (14.9)			
Preobese	26 (38.8)	21 (31.3)			
Obese	6 (9)	8 (11.9)			
Personal History, n (%)					
Smoking	20 (29.8)	23 (34.3)	Chi-square test	12.093	0.65
Alcohol	18 (26.8)	25 (37.3)			
Betel nut	6 (8.9)	6 (8.9)			
Comorbidities, n (%)					
Diabetes	18 (26.8)	17 (25.3)	Chi-square test	34.039	0.495
Hypertension	20 (29.8)	16 (23.8)			
Hypothyroidism	12 (17.9)	10 (14.9)			
Duration of illness at presentation					
Mean ± SD	6.35 ± 2.98				

Classical lichen planus was the most common morphological type noted followed by hypertrophic lichen planus, lichen planus pigmentosus, and oral lichen planus. Oral lichen planus and lichen planus pigmentosus were more common in males than in females, in contrast to other types (Table 2).

**Table 2:** Morphological types of lichen planus among the patients

Diagnosis	No. of cases, n (%)
Classic Lichen Planus	36 (53.7%)
Eruptive Lichen Planus	3 (4.5%)
Follicular Lichen Planus	1 (1.5%)
Hypertrophic Lichen Planus	7 (10.4%)
Lupus Erythematosus - Lichen Planus overlap	1 (1.5%)
Lichen Plano Pilaris	2 (3.0%)
Lichen Planus Pigmentosus	7 (10.4%)
Linear Lichen Planus	2 (3.0%)
Nail Lichen Planus	1 (1.5%)
Oral Lichen Planus	7 (10.4%)
Total	67 (100%)

Oral involvement was seen in 21 patients. According to the Andreasen Classification, among the seven patients with only oral lichen planus, four patients had the reticular type, and the papular, erosive, and plaque types were seen in one patient each. Among these patients, two had dental amalgam fillings. 14 patients had cutaneous lichen planus with oral involvement, 10 patients had classical lichen planus, two had hypertrophic lichen planus, and two had lichen planus pigmentosus.

Nail involvement was noted in 11 patients. Longitudinal melanonychia was the most common nail change noted, followed by pitting, subungual hyperkeratosis, pterygium, and dystrophy. Koebnerisation was seen in 15 (22.3%) of patients.

Overall, LP patients had a higher prevalence of dyslipidemia, 41 (67%), compared to the controls, 38 (56.7%), although there was no statistical significance (p-value = 0.783). However, on considering individual parameters, LP patients had significantly higher levels of total cholesterol and LDL compared to controls. HDL levels were similar between both groups. Triglyceride levels were higher in cases compared to controls but showed statistical significance only in males (Table 3).

**Table 3:** Comparing lipid profiles between case and control groups *p<0.05 considered significant

	Patient group	Control group	t-test statistic value	p-value
Total cholesterol				
Overall	198.07 ± 31.7	136.41 ± 36.47	0.434	<0.001*
Females	200.91 ± 29.97	181.64 ± 26.25	0.498	0.011*
Males	193.93 ± 34.74	154.25 ± 40.07	0.786	0.001*
Serum triglycerides				
Overall	136.36 ± 44.12	124.19 ± 46.16	0.138	0.138
Females	142.22 ± 48.35	139.17 ± 43.69	0.231	0.772
Males	140.32 ± 47.82	113.97 ± 46.27	0.864	0.040*
High density lipoprotein				
Overall	40.49 ± 9.37	39.42 ± 9.89	0.595	0.534
Females	40.55 ± 9.21	41.41 ± 7.5	0.936	0.709
Males	39.45 ± 8.75	36.38 ± 11.18	0.537	0.204
Low density lipoprotein				
Overall	131.73 ± 32.11	107.33 ± 25.16	0.603	<0.001*
Females	134.69 ± 33.89	115.03 ± 18.99	0.589	0.006*
Males	131.94 ± 32.21	98.29 ± 28.48	0.536	<0.001*

**Figure 1:**
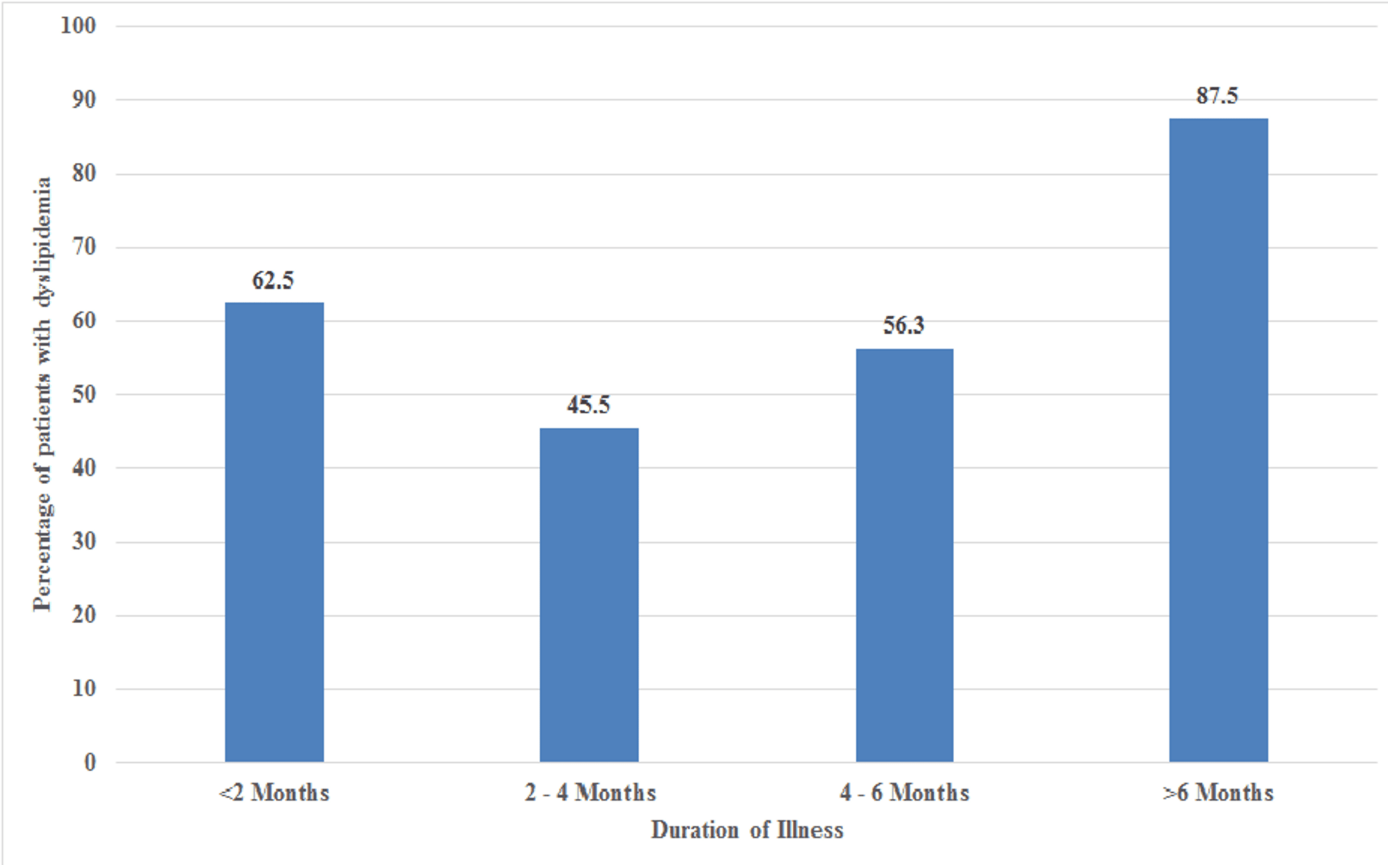
Percentage of patients with dyslipidemia and the duration of illness at presentation

Patients with a duration of illness of more than 6 months had a significantly higher prevalence of dyslipidemia compared to patients with a shorter duration of illness (p-value = 0.043) (Figure 1).

## Discussion

Lichen planus is a chronic, immune-mediated inflammatory disease characterized by increased levels of inflammatory cytokines in the skin lesions. There is a well-established link between the cytokines TNF-α, IL-6, IL-10, and IL-4 and lipid metabolism disturbances. Dyslipidemia can have serious consequences such as atherosclerosis which leads to coronary artery disease, cerebrovascular accidents, and peripheral vascular diseases. It can also cause pancreatitis and hepatosplenomegaly. Hence, this study was conducted to further explore the link between lichen planus and dyslipidemia [1-4].

The prevalence of hypertension, diabetes, and hypothyroidism was lower in our LP study population compared to other studies [6-8]. This difference likely reflects variations in cultural practices, lifestyle, and the younger average age of our study participants. Our findings regarding dyslipidemia prevalence were compared with those of prior studies. While our study, along with those by Azeez et al. [6], Arshad et al. [7], and Ozkur et al. [8], indicated a slightly higher prevalence of dyslipidemia compared to controls, these differences were not statistically significant. In contrast, Mathur et al. [9] reported a statistically significant higher prevalence of dyslipidemia (p=0.001) among lichen planus patients (Table 4).

**Table 4:** Comparison of prevalence of dyslipidemia among various studies

Study	Cases (%)	Control (%)
Azeez et al. [6]	63.8%	48.9%
Arshad et al. [7]	57.9%	56.8%
Ozkur et al. [8]	67.3%	64.6%
Mathur et al. [9]	60%	34%
Present Study	61.2%	56.7%

An analysis of individual lipid parameters in cases versus controls across multiple studies suggests a potential association between elevated total cholesterol and LDL levels and the presence of LP, consistent with our findings. However, discrepancies persist in the literature, particularly concerning HDL and triglyceride levels. In our study, although triglyceride levels were elevated in both groups, statistical significance was observed only among male patients. This contrasts with findings from other studies [8, 10, 11] (Table 5), which reported significant increases in triglyceride levels in both male and female patients. These differences may be attributed to a variety of factors, including variations in dietary habits, patterns of alcohol consumption, and the influence of hormonal factors, which are known to generally contribute to lower triglyceride levels in females.

**Table 5:** Comparing lipid parameters among various studies HDL: high-density lipoprotein; LDL: low-density lipoprotein

Cases/Control	Variable, mg/dl (Mean ± SD)	Ozkur et al. [8]	Ozbagcivan et al. [10]	Kuntoji et al. [11]	Present Study
Cases	Total Cholesterol	202.9±47.2	219.77 ± 43.61	158.49±33.91	198.07± 31.7
	HDL	51.2±14.4	50.43±7.60	38.86±10.49	40.49±9.37
	LDL	127.8±36.7	131.47±34.95	88.74±30.46	131.73±32.11
	Triglyceride	136.6±95.8	138.07±54.89	153.03±99.77	136.36±44.12
Control	Total Cholesterol	193.5±48.8	192.13±36.02	143.47±28.54	136.41±36.47
	HDL	47.9±14.4	57.13±14.16	45.78±7.63	39.42±9.89
	LDL	119.4±41.0	113.37±29.27	79.02±20.17	107.33±25.16
	Triglyceride	124.3±58.1	93.80±31.00	107.91±62.38	124.19±46.16

Further supporting the lipid profile alterations in LP, Gonzalez Navarro B et al.'s systematic review has demonstrated a significant association between dyslipidemia and oral lichen planus (OLP), particularly in its erosive and reticular forms [12]. In this study, dyslipidemia was observed in 12 of 21 patients (57%) with oral lichen planus, implying a possible association. However, the limited sample size precluded the assessment of statistical significance. Nonetheless, these findings suggest that oral involvement in LP may serve as a useful clinical marker for increased dyslipidemia risk, underscoring the importance of metabolic screening in patients presenting with OLP.

Another noteworthy observation from our study was that patients with a disease duration exceeding six months exhibited a significantly higher prevalence of dyslipidemia compared to those with a shorter duration of illness (p = 0.043). This finding suggests a possible cumulative effect of chronic inflammation associated with lichen planus on lipid metabolism, further reinforcing the need for early metabolic assessment and long-term monitoring in affected individuals.

A limitation of the study is that it does not rule out the possibility that some patients may have experienced dyslipidemia prior to developing LP since it is a cross-sectional design which prevents any conclusions regarding the temporal relationship between LP and dyslipidemia. Another limitation is that the study was conducted at a single center with a limited sample size. Further research with larger sample sizes, longer duration, and standardized methodologies is warranted to clarify the precise relationship between lipid parameters and LP.

## Conclusions

In our study, significantly higher total cholesterol and LDL levels in both male and female lichen planus (LP) patients, and elevated triglycerides in males were noted. However, the overall prevalence of dyslipidemia did not differ significantly. This apparent paradox suggests a potential association between the chronic inflammatory state of LP and disruptions in lipid metabolism. The elevated lipid levels and comorbidities highlight the necessity for integrated management, addressing cardiovascular risk factors alongside dermatological aspects.
